# Searching and Tracking an Unknown Number of Targets: A Learning-Based Method Enhanced with Maps Merging

**DOI:** 10.3390/s21041076

**Published:** 2021-02-04

**Authors:** Peng Yan, Tao Jia, Chengchao Bai

**Affiliations:** 1 School of Astronautics, Harbin Institute of Technology, Harbin 150001, China; yanpeng@hit.edu.cn; 2 Aerospace Technology Research Institute, China Aerodynamics Research and Development Center, Mianyang 621000, China; 13946034815@163.com

**Keywords:** unmanned aerial vehicle (UAV), search and track, deep reinforcement learning (DRL), maps merging, convolutional neural network (CNN)

## Abstract

Unmanned aerial vehicles (UAVs) have been widely used in search and rescue (SAR) missions due to their high flexibility. A key problem in SAR missions is to search and track moving targets in an area of interest. In this paper, we focus on the problem of Cooperative Multi-UAV Observation of Multiple Moving Targets (CMUOMMT). In contrast to the existing literature, we not only optimize the average observation rate of the discovered targets, but we also emphasize the fairness of the observation of the discovered targets and the continuous exploration of the undiscovered targets, under the assumption that the total number of targets is unknown. To achieve this objective, a deep reinforcement learning (DRL)-based method is proposed under the Partially Observable Markov Decision Process (POMDP) framework, where each UAV maintains four observation history maps, and maps from different UAVs within a communication range can be merged to enhance UAVs’ awareness of the environment. A deep convolutional neural network (CNN) is used to process the merged maps and generate the control commands to UAVs. The simulation results show that our policy can enable UAVs to balance between giving the discovered targets a fair observation and exploring the search region compared with other methods.

## 1. Introduction

In the past decade, unmanned aerial vehicles (UAVs) have been widely used in military and civilian applications due to their low cost and high flexibility. Especially in search and rescue (SAR) missions, multiple UAVs working together can reduce mission execution time and provide timely relief to targets [1,2,3]. In a SAR mission, UAVs need to search out targets in an unknown region and continuously track them to monitor their status. However, in general, the number of targets is unknown and available UAVs are limited, which requires multiple UAVs to work together to keep track of the discovered targets while finding more unknown targets [4]. The problem of using robot teams to cooperatively observe multiple moving targets has been formalized first by Parker and Emmons [5], who termed this problem as Cooperative Multi-Robot Observation of Multiple Moving Targets (CMOMMT) and showed it is NP-hard.

Since the CMOMMT problem was raised, there has been a great deal of work to address it. A classical approach is the local force vector proposed by Parker and Emmons [5], in which a robot is subject to the attractive forces of nearby targets and the repulsive forces of nearby robots, and the direction of the robot’s motion is determined by the combined force of the two. However, this method will cause overlapping observations on the same target. Thus, Parker [6] proposed an improved method called A-CMOMMT to solve this phenomenon, where the robots are controlled by the weighted local force vectors for tracking targets. Additionally, in [7], the authors proposed B-CMOMMT, in which a help behavior is added to reduce the risk of losing a target. In [8], the authors proposed an algorithm called P-CMOMMT, considering the uniformity of the observation of the targets through the information entropy of targets’ observations. The methods based on local force vector lack the prediction of the targets’ behaviors and do not make full use of the targets’ historical position information, resulting in a low efficiency for searching and tracking the targets.

A large number of optimization-based methods have been proposed to solve CMOMMT-like problems [9,10,11,12]. In [13], the authors used group of vision-based UAVs to search for multiple ground targets. The objective is to optimize the collective coverage area and the detection performance based on a distributed probability map updating model, in which the probability of target existence is calculated through the measurement information and information sharing among neighboring agents. In [14], the authors proposed a multi-objective optimization approach based on genetic algorithm (GA) to minimize the mission completion time for a team of UAVs finding a target in a bounded area. In [15], the UAVs’ task sequence for a reconnaissance task assignment problem is considered, where the problem is formulated as a multi-objective, multi-constraint, nonlinear optimization problem solved with a modified Multi-Objective Symbiotic Organisms Search algorithm (MOSOS). In [16], searching and tracking an unknown ground moving target by multiple UAVs in an urban environment was modeled as a multi-objective optimization problem with preemptive priority constraints. The authors proposed a fuzzy multi-objective path planning method to solve this problem with target behavior predicted by extended Kalman filter (EKF) and probability estimation. In [17], the authors proposed a real-time path-planning solution enabling multiple UAVs to cooperatively search a given area. The problem is modeled as a Model Predictive Control (MPC) problem solved with Particle Swarm Optimization (PSO) algorithm. In [18], the authors emphasize the fairness of observations among different targets compared with the initial CMOMMT problem. They proposed an integer linear programming model to solve this problem where the motion of the targets is estimated in a Bayesian framework.

The above-mentioned approaches fail to balance between target searching and target tracking, which will make it difficult for UAVs to keep searching for undiscovered targets when the number of UAVs is less than the number of targets. To solve this problem, Li et al. [19] proposed a profit-driven adaptive moving targets search algorithm, which considers the impact of moving targets and collaborating UAVs in a unified framework through a concept called observation profit of cells. However, this approach assumes that the total number of targets is known, which is impractical in some complex environments. In [20], Dames proposed a method to enable multiple robots to search for and track an unknown number of targets. The robots use the Probability Hypothesis Density (PHD) filter to estimate the number of targets and the positions of the targets, and a Voronoi-based control strategy to search and track targets. This method assumes that each robot has a unique ID for creating a globally consistent estimate, which will limit the scalability of the robot team.

Recently, the development of deep reinforcement learning (DRL) [21] provides an alternative way to deal with the CMOMMT problem. DRL learns control policies through interacting with the environment, and it has reached or exceeded human levels in some game tasks [22,23]. There have been some studies using DRL to solve the targets search and tracking problem. In [24], the authors proposed a framework for searching for multiple static targets through a group of UAVs. The framework consists of a global planner based on a modern online Partially Observable Markov Decision Process (POMDP) solver and a local continuous-environment exploration controller based on a DRL method. In [25], the authors proposed a target following method based on deep Q-networks, considering visibility obstruction from obstacles and uncertain target motion. In [26], the authors proposed a DRL-based method to enable a robot to explore unknown cluttered urban environments, in which a deep network with convolutional neural network (CNN) [27] was trained by asynchronous advantage actor-critic (A3C) approach to generate appropriate frontier locations. In [28], the authors constructed a framework for automatically exploring unknown environments. The exploration process is decomposed into the decision, planning, and mapping modules, in which the decision module is implemented by a deep Q-network for learning exploration policy from the partial map.

In this paper, we focus on the problem of Cooperative Multi-UAV Observation of Multiple Moving Targets (CMUOMMT), where a UAV team needs to search and track an unknown number of targets in a search region. Our objective is to enable UAVs to give the discovered targets a fair observation and meanwhile maximize the exploration rate of the environment to discover more targets. To achieve this objective, the problem is formulated as a POMDP and solved with a DRL method. During the mission, each UAV maintains four observation history maps, which can reduce the partial observability of the environment. Furthermore, maps merging among UAVs can further improve awareness of the environment. To extract environmental features, a deep network with CNN is used to process each UAV observation map. A modern DRL method is used to train the shared policy with a centralized training, decentralized execution paradigm. The main contributions of this work are as follows:

- The average observation rate of the targets, the standard deviation of the observation rates of the targets, and the exploration rate of the search region are simultaneously optimized to enable multiple UAVs to cooperatively achieve fair observation of discovered targets and continuous search for undiscovered targets.
- Each UAV maintains four observation maps recording observation histories, and a map merging method among UAVs is proposed, which can reduce the partial observability of the environment and improve awareness of the environment.
- A DRL-based multi-UAV control policy is proposed, which allows UAVs to learn to balance tracking targets and exploring the environment by interacting with the environment.

The remainder of this paper is organized as follows. In Section 2, the problem is formulated and the optimization objectives are introduced. In Section 3, the details of our method are proposed, including the maps merging method and the key ingredients of the DRL method. In Section 4, simulation experiments are conducted and the results are discussed. Finally, we conclude this paper in Section 5.

## 2. Problem Formulation

**Figure 1:**
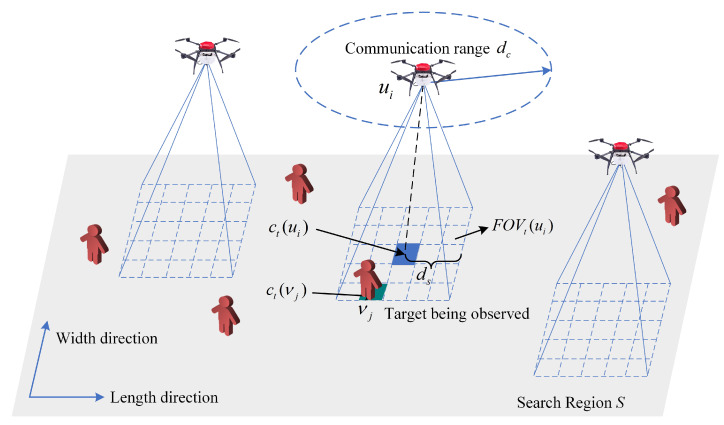
The environment considered in this paper. Unmanned aerial vehicles (UAVs) and targets are moving in a bounded two-dimensional rectangular search region *S*. $c_{t}\left(\nu_{j}\right)$ and $c_{t}\left(u_{i}\right)$ denote the cells in which target $\nu_{j}$ and UAV $u_{i}$ lie at time step *t*, respectively. The blue grids represent the FOV (field of view) of each UAV. The dashed ellipse indicates the communication range of the UAV $u_{i}$.

In this paper, we consider the problem of CMUOMMT described in [6,19], which is shown in Figure 1 and defined as follows:

- A bounded two-dimensional rectangular search region *S* discretized into $C_{L}\times C_{W}$ equally sized cells, where $C_{L}$ and $C_{W}$ represent the number of cells in the length and width directions of the search region, respectively.
- The time step is discretized and denoted by *t* within a mission time duration *T*.
- A set of *N* moving targets V in *S*. For target $\nu_{j}\left(\nu_{j}\in V,j=1,2,\cdots N\right)$, the cell that lies at time step *t* is denoted by $c_{t}\left(\nu_{j}\right)\in S$. The mission is to observe these targets using multiple UAVs. To simplify this mission, we assume that the maximal speed of the targets is smaller than that of the UAVs.
- A team of *M* homogeneous UAVs U deployed in *S* to observe the targets. For UAV $u_{i}\left(u_{i}\in U,\;i=1,2,\cdots M\right)$, the cell that lies at time step *t* is denoted by $c_{t}\left(u_{i}\right)\in S$. Each UAV can observe the targets through its onboard sensor. The sensing range of each UAV is denoted by $d_{s}$. We assume that the UAVs are flying at a fixed altitude, and the size of the field of view (FOV) of each UAV is the same and remains constant. The term $FOV_{t}\left(u_{i}\right)$ denotes the FOV of the UAV $u_{i}$ at time step *t*. In addition, each UAV is equipped with a communication device to share information to coordinate with other UAVs. The communication range is denoted by $d_{c}$, which is assumed to be larger than the sensing range $d_{s}$. The UAVs can only share information with UAVs within a communication range. We further assume that all UAVs share a known global coordinate system.

The target $\nu_{j}$ is monitored when it is within the FOV of at least one UAV, which can be defined as

(1) $$O_{t}\left(\nu_{j}\right)=\left\{\begin{array}{c} 1,\mathrm{if}\;\exists u_{i},c_{t}\left(\nu_{j}\right)\in FOV_{t}\left(u_{i}\right)\\ 0,\mathrm{else} \end{array}\right.$$

where $O_{t}\left(\nu_{j}\right)$ indicates the observation state of target $\nu_{j}$.

During the mission, the observation rate of the target $\nu_{j}$ can be defined as

(2) $$\eta \left(\nu_{j}\right)=\frac{1}{T}\sum_{t=1}^{T}O_{t}\left(\nu_{j}\right)$$

where $\eta (\nu_{j})$ represents the observation rate of the target $\nu_{j}$, which represents the proportion of time elapsed under the observation of at least one UAV during the mission.

The first objective for the UAV team is to maximize the average observation rate of *N* targets, which can be characterized by the metric $\bar{\eta }$:

(3) $$\bar{\eta }=\frac{1}{N}\sum_{j=1}^{N}\eta (\nu_{j})$$

Maximizing $\bar{\eta }$ alone is unfair, especially when the number of UAVs is less than the number of targets, which may result in some targets not being observed during the mission. To solve this problem, the second objective for the UAV team is to minimize the standard deviation $\sigma_{\eta }$ of the observation rates of *N* targets:

(4) $$\sigma_{\eta }=\sqrt{\frac{1}{N}\sum_{j=1}^{N}\left(\eta \left(\nu_{j}\right)-\bar{\eta }\right)}$$

A low value of $\sigma_{\eta }$ means that all targets are observed relatively uniformly during the mission. In addition, since the UAV team does not know the total number of targets, it needs to continuously explore the search region to discover new targets. Thus, the third objective for the UAV team is to maximize the exploration rate β of the search region, which is defined as

(5) $$\beta =\frac{1}{T}\frac{1}{C_{L}C_{W}}\sum_{k=1}^{C_{L}}\sum_{l=1}^{C_{W}}t_{stamp}\left(c_{kl}\right)$$

where $t_{stamp}\left(c_{kl}\right)$ represents the latest observed time for cell $c_{kl}$. *k* and *l* represent the indexes of the cell $c_{kl}$ in the length and width directions of the search region, respectively. The maximum value of β is 1, which means that all cells in the search region are being observed by the UAV team at time step *T*. However, this is unrealistic since the maximum region observed by the UAV team is less than the total search region to be observed. That is,

(6) $$\underset{u_{i}\in U}{\cup }FOV\left(u_{i}\right)<S$$

The ultimate objective is a combination of $\bar{\eta }$, $\sigma_{\eta }$ and β, which is different from [6,19], whose objectives only consider the average observation rate $\bar{\eta }$. In this study, the UAV team needs to balance between giving the known targets a fair observation and exploring the search region through an efficient method.

## 3. Methods

### 3.1. Overview

We formulate the CMUOMMT problem as a POMDP and solve it with a DRL method. In this method, all UAVs share a global control policy π to decide actions. The action is selected according to the observation from the environment, i.e., $a_{t}∼\pi \left(a_{t}|o_{t}\right)$, $o_{t}∼O\left(s_{t}\right)$, where $s_{t}$ is the global state of the environment, $o_{t}$ is the local observation of the environment state, $O(s_{t})$ is the observation function determined by the UAVs’ sensing range and communication range, and $a_{t}$ is the selected action. The observation $o_{t}$ includes four observation maps about the environment, which will be given in Section 3.2.

In a reinforcement learning (RL) framework, an RL agent learns an optimal policy $a_{t}∼\pi^{*}\left(a_{t}|o_{t}\right)$ through interacting with the environment. The goal of the RL agent is to maximize a long-term accumulated reward

(7) $$G_{t}=\sum_{k=0}^{\infty }\gamma^{k}r_{t+k+1}$$

where $r_{t+k+1}$ is the reward the RL agent received at time step t+k+1, γ(0<γ<1) is the discount factor to make $G_{t}$ a bounded value.

**Figure 2:**
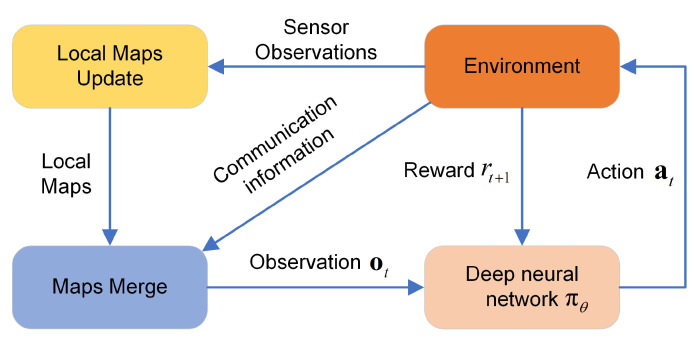
The system architecture.

In the proposed DRL method, we use a deep neural network $\pi_{\theta }\left(a_{t}|o_{t}\right)$ parameterized by θ to approximate the UAVs’ control policy. The objective is to use a DRL method to find the optimal parameters $\theta^{*}$, which can make the UAV team balance between giving the known targets a fair observation and exploring the search region. The system architecture is shown in Figure 2.

As shown in Figure 2, each UAV first gets the observations from the environment through its onboard sensor to update its local observation maps. Then, each UAV receives the local maps of the other UAVs through its communication device, and the local maps are merged to provide the deep network $\pi_{\theta }$ an observation $o_{t}$. Finally, the deep neural network $\pi_{\theta }$ outputs the action $a_{t}$ to control the UAV and receives the reward $r_{t+1}$ at the next time step. The maps merging method is introduced in the next subsection, and the ingredients of deep reinforcement learning are introduced in Section 3.3.

### 3.2. Maps Merging

During the mission, each UAV maintains four observation maps:

(1) The observation map of the UAV’s position in the search region, denoted by a $C_{L}\times C_{W}$ matrix $MS_{t}\left(u_{i}\right)$, $MS_{t}\left(u_{i}\right)\in R^{2}$, defined as follows:

(8) $$MS_{t}\left(u_{i}\right)={\left[ms_{t}^{kl}\left(u_{i}\right)\right]}_{C_{L}\times C_{W}},\;ms_{t}^{kl}\left(u_{i}\right)=\left\{\begin{array}{c} 1,\;\mathrm{if}\;c_{t}\left(u_{i}\right)\;=\;c_{kl}\\ 0,\;\mathrm{else} \end{array}\right.$$

(2) The observation history map of the cells, which records the latest observed time for each cell. This map is denoted by a $C_{L}\times C_{W}$ matrix $MC_{t}\left(u_{i}\right)$, $MC_{t}\left(u_{i}\right)\in R^{2}$, defined as follows:

(9) $$MC_{t}\left(u_{i}\right)={\left[mc_{t}^{kl}\left(u_{i}\right)\right]}_{C_{L}\times C_{W}}$$

The map $MC_{t}\left(u_{i}\right)$ is obtained in two steps. At each time step *t*, the map $MC_{t}\left(u_{i}\right)$ is first updated by the observation of the UAV $u_{i}$ on the subset of cells within $FOV_{t}\left(u_{i}\right)$, that is,

(10) $$mc_{t}^{kl}\left(u_{i}\right)=t,\;\;\mathrm{for}\;\;c_{kl}\in FOV_{t}\left(u_{i}\right)$$

In addition, the observation history maps from other UAVs within a communication range will also update the local maps. The values of corresponding cells in the observation history map will be updated with the latest observation time as follows:

(11) $$mc_{t}^{kl}\left(u_{i}\right)=mc_{t}^{kl}\left(u_{j}\right),\;\mathrm{if}\;mc_{t}^{kl}\left(u_{j}\right)>mc_{t}^{kl}\left(u_{i}\right)\;\mathrm{and}\;d_{t}\left(u_{i},\;u_{j}\right)<d_{c},\;u_{j}\in U,\;j\neq i$$

where $d_{t}\left(u_{i},u_{j}\right)$ represents the distance between UAV $u_{i}$ and UAV $u_{j}$.

(3) The position history map of the other UAVs, which records the history positions of the other UAVs. This map is denoted by a $C_{L}\times C_{W}$ matrix $MU_{t}\left(u_{i}\right)$, $MU_{t}\left(u_{i}\right)\in R^{2}$, defined as follows:

(12) $$MU_{t}\left(u_{i}\right)={\left[mu_{t}^{kl}\left(u_{i}\right)\right]}_{C_{L}\times C_{W}},$$

(13) $$mu_{t}^{kl}\left(u_{i}\right)=\left\{\begin{array}{c} 1,\;\mathrm{if}\;c_{t}\left(u_{j}\right)\;=c_{kl}\;\mathrm{and}\;d_{t}\left(u_{i},\;u_{j}\right)<d_{c},\;u_{j}\in U,\;i\neq j\\ 0,\;\mathrm{if}\;t=0 \end{array}\right.$$

At each time step, the map $MU_{t}\left(u_{i}\right)$ is updated by the observation history maps from other UAVs within a communication range as follows:

(14) $$mu_{t}^{kl}\left(u_{i}\right)=mu_{t}^{kl}\left(u_{j}\right),\;\mathrm{if}\;mu_{t}^{kl}\left(u_{j}\right)>mu_{t}^{kl}\left(u_{i}\right)\;\mathrm{and}\;d_{t}\left(u_{i},\;u_{j}\right)<d_{c},\;u_{j}\in U,\;j\neq i$$

In addition, the knowledge about the positions of the other UAVs at the last observation time might be outdated. Thus, at the next time step, the values of cells in $MU_{t}\left(u_{i}\right)$ are decayed as follows:

(15) $$mu_{t+1}^{kl}\left(u_{i}\right)=mu_{t}^{kl}\left(u_{i}\right)-\frac{1}{t_{U}},\;\;\mathrm{if}\;\;mu_{t}^{kl}\left(u_{i}\right)\geq \frac{1}{t_{U}}$$

where $t_{U}$ is a time constant, representing the decay period of the value of $mu_{t}^{kl}\left(u_{i}\right)$.

(4) The position history map of the targets, which records the historical positions of the targets. This map is denoted by a $C_{L}\times C_{W}$ matrix $MT_{t}\left(u_{i}\right)$, $MT_{t}\left(u_{i}\right)\in R^{2}$, defined as follows:

(16) $$MT_{t}\left(u_{i}\right)={\left[mt_{t}^{kl}\left(u_{i}\right)\right]}_{C_{L}\times C_{W}},$$

(17) $$mt_{t}^{kl}\left(u_{i}\right)=\left\{\begin{array}{c} 1,\;\mathrm{if}\;c_{t}\left(\nu_{j}\right)=c_{kl}\;\mathrm{and}\;c_{t}\left(\nu_{j}\right)\in FOV_{t}\left(u_{i}\right),\;\nu_{j}\in V\\ 0,\;\mathrm{if}\;t=0 \end{array}\right.$$

The map $MT_{t}\left(u_{i}\right)$ is also updated by the observation history maps from other UAVs within a communication range, that is,

(18) $$mt_{t}^{kl}\left(u_{i}\right)=mt_{t}^{kl}\left(u_{j}\right),\;\mathrm{if}\;mt_{t}^{kl}\left(u_{j}\right)>mt_{t}^{kl}\left(u_{i}\right)\;\mathrm{and}\;d_{t}\left(u_{i},\;u_{j}\right)<d_{c},\;u_{j}\in U,\;j\neq i$$

Same as the map $MU_{t}\left(u_{i}\right)$, the values of cells in $MT_{t}\left(u_{i}\right)$ are decayed as follows:

(19) $$mt_{t+1}^{kl}\left(u_{i}\right)=mt_{t}^{kl}\left(u_{i}\right)-\frac{1}{t_{T}},\;\;\mathrm{if}\;\;mt_{t}^{kl}\left(u_{i}\right)\geq \frac{1}{t_{T}}$$

where $t_{T}$ is a time constant, representing the decay period of the value of $mt_{t}^{kl}\left(u_{i}\right)$.

**Figure 3:**
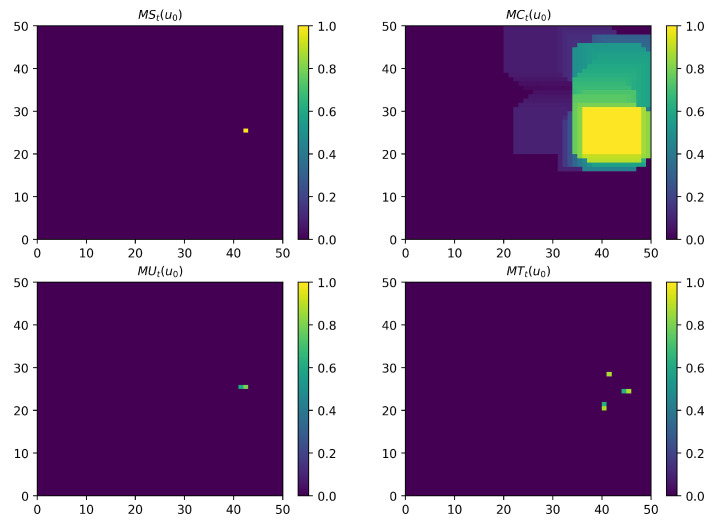
An illustration of four observation maps for UAV $u_{0}$. The parameters are set as follows: N=10, M=5, $C_{L}=50$, $C_{W}=50$, $d_{s}=5\;\mathrm{cells}$, $d_{c}=10\;\mathrm{cells}$, $t_{U}=5$, $t_{T}=8$. The current time step is t=200. The map $MC_{t}\left(u_{0}\right)$ is normalized as follows: $mc_{t}^{kl}\left(u_{0}\right)=mc_{t}^{kl}\left(u_{0}\right)/\max\left(MC_{t}\left(u_{0}\right)\right)$, where $\max(MC_{t}\left(u_{0}\right))$ represents the maximum value of elements in matrix $MC_{t}\left(u_{0}\right)$. From the map $MU_{t}\left(u_{0}\right)$, we can see that this map records one UAV’s historical positions. From the map $MT_{t}\left(u_{0}\right)$, we can see that this map records three targets’ historical positions.

One example of four observation maps for UAV $u_{0}$ is shown in Figure 3.

### 3.3. Deep Reinforcement Learning

In this section, we introduce the key elements of the proposed DRL method, consisting of observation space, action space, network architecture, reward function, and training algorithm.

#### 3.3.1. Observation Space

At time step *t*, the observation of UAV $u_{i}$ consists of four parts, i.e., $o_{t}\left(u_{i}\right)=\left[o_{t}^{1}\left(u_{i}\right),\;o_{t}^{2}\left(u_{i}\right),\;o_{t}^{3}\left(u_{i}\right),\;o_{t}^{4}\left(u_{i}\right)\right]$.

- The observation $o_{t}^{1}\left(u_{i}\right)$ is a part of the map $MS_{t}\left(u_{i}\right)$ centered in the UAV’s current cell $c_{t}\left(u_{i}\right)$, with length $C_{\mathrm{input}}$ and width $C_{\mathrm{input}}$. That is, $o_{t}^{1}\left(u_{i}\right)$ is a $C_{\mathrm{input}}\times C_{\mathrm{input}}$ matrix, representing the positional relationship of UAV $u_{i}$ relative to the boundary of the search area *S*, which is defined as follows:
  (20) $$o_{t}^{1}\left(u_{i}\right)={\left[{}^{1}o_{t}^{kl}\left(u_{i}\right)\right]}_{C_{\mathrm{input}}\times C_{\mathrm{input}}},$$
  (21) $${}^{1}o_{t}^{kl}\left(u_{i}\right)=\left\{\begin{array}{c} 1,\;\mathrm{if}\;c_{t}\left(u_{i}\right)\;=c_{kl}\;\mathrm{or}\;c_{kl}\notin S,k=1,2,\cdots C_{\mathrm{input}},\\ \;l=1,2,\cdots C_{\mathrm{input}}\\ 0,\;\mathrm{else} \end{array}\right.$$
- The observation $o_{t}^{2}\left(u_{i}\right)$ is a part of the map $MC_{t}\left(u_{i}\right)$ centered in the UAV’s current cell $c_{t}\left(u_{i}\right)$, with length $C_{\mathrm{input}}$ and width $C_{\mathrm{input}}$. Similarly, $o_{t}^{2}\left(u_{i}\right)$ is a $C_{\mathrm{input}}\times C_{\mathrm{input}}$ matrix, representing the observation state of the cells around UAV $u_{i}$, which is defined as follows:
  (22) $$o_{t}^{2}\left(u_{i}\right)={\left[{}^{2}o_{t}^{kl}\left(u_{i}\right)\right]}_{C_{\mathrm{input}}\times C_{\mathrm{input}}},$$

(23) $${}^{2}o_{t}^{kl}\left(u_{i}\right)=\left\{\begin{array}{c} mc_{t}^{mn}\left(u_{i}\right)/t,\;\mathrm{if}\;t>0\;\mathrm{and}\;c_{kl}\in S\;\mathrm{and}\;c_{kl}=c_{mn}\;,k=1,2,\cdots C_{\mathrm{input}},\\ \;l=1,2,\cdots C_{\mathrm{input}},\;m=1,2,\cdots C_{L},\;n=1,2,\cdots C_{W}\\ 1,\;\mathrm{elseif}\;c_{kl}\notin S\\ 0,\;\mathrm{else} \end{array}\right.$$

- The observation $o_{t}^{3}\left(u_{i}\right)$ is a part of the map $MU_{t}\left(u_{i}\right)$ centered in the UAV’s current cell $c_{t}\left(u_{i}\right)$, with length $C_{\mathrm{input}}$ and width $C_{\mathrm{input}}$. Like $o_{t}^{2}\left(u_{i}\right)$, $o_{t}^{3}\left(u_{i}\right)$ is a $C_{\mathrm{input}}\times C_{\mathrm{input}}$ matrix, representing historical position information of other UAVs around UAV $u_{i}$, which is defined as follows:
  (24) $$o_{t}^{3}\left(u_{i}\right)={\left[{}^{3}o_{t}^{kl}\left(u_{i}\right)\right]}_{C_{\mathrm{input}}\times C_{\mathrm{input}}},$$

(25) $${}^{3}o_{t}^{kl}\left(u_{i}\right)=\left\{\begin{array}{c} mu_{t}^{mn}\left(u_{i}\right),\;\mathrm{if}\;c_{kl}\in S\;\mathrm{and}\;c_{kl}=c_{mn},\;k=1,2,\cdots C_{\mathrm{input}},\\ \;l=1,2,\cdots C_{\mathrm{input}},\;m=1,2,\cdots C_{L},\;n=1,2,\cdots C_{W}\\ 0,\;\mathrm{else} \end{array}\right.$$

- The observation $o_{t}^{4}\left(u_{i}\right)$ is a part of the map $MT_{t}\left(u_{i}\right)$ centered in the UAV’s current cell $c_{t}\left(u_{i}\right)$, with length $C_{\mathrm{input}}$ and width $C_{\mathrm{input}}$. Similarly, $o_{t}^{4}\left(u_{i}\right)$ is a $C_{\mathrm{input}}\times C_{\mathrm{input}}$ matrix, representing historical position information of targets around UAV $u_{i}$, which is defined as follows:
  (26) $$o_{t}^{4}\left(u_{i}\right)={\left[{}^{4}o_{t}^{kl}\left(u_{i}\right)\right]}_{C_{\mathrm{input}}\times C_{\mathrm{input}}},$$

(27) $${}^{4}o_{t}^{kl}\left(u_{i}\right)=\left\{\begin{array}{c} mt_{t}^{mn}\left(u_{i}\right),\;\mathrm{if}\;c_{kl}\in S\;\mathrm{and}\;c_{kl}=c_{mn},\;k=1,2,\cdots C_{\mathrm{input}},\\ \;l=1,2,\cdots C_{\mathrm{input}}\;,m=1,2,\cdots C_{L},\;n=1,2,\cdots C_{W}\\ 0,\;\mathrm{else} \end{array}\right.$$

**Figure 4:**
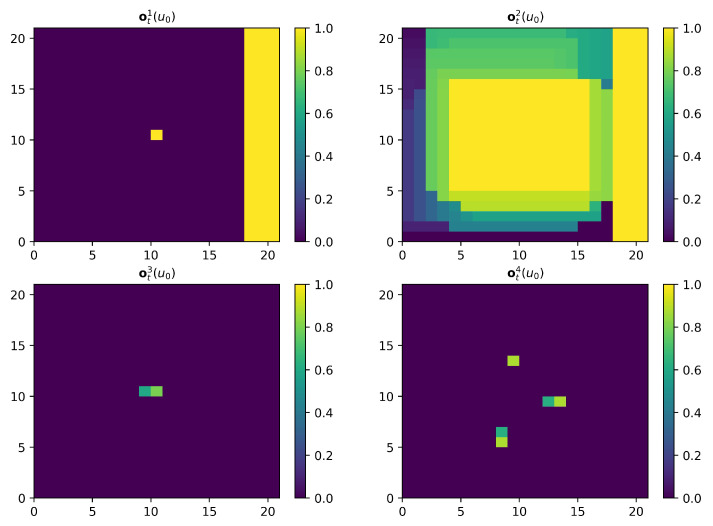
An illustration of observations for UAV $u_{0}$. The parameters are consistent with those in Figure 3. The value of $C_{\mathrm{input}}$ is set to 21 cells. From the observation $o_{t}^{1}\left(u_{0}\right)$ and $o_{t}^{2}\left(u_{0}\right)$, we can see that UAV $u_{0}$ is very close to the right boundary of the search region.

One example of observations for UAV $u_{0}$ is shown in Figure 4, which is consistent with the scenario shown in Figure 3.

#### 3.3.2. Action Space

The UAV’s action space is a set of target cells around the UAV, that is, each UAV can move into one of its eight neighbor cells or stay at its current cell. Thus, the action space has a total of nine command actions. The actual command action is selected according to the selection probabilities calculated by the deep neural network.

#### 3.3.3. Network Architecture

**Figure 5:**
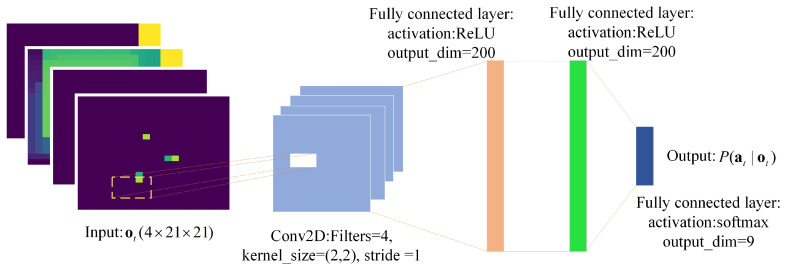
The deep neural network architecture. The value of $C_{\mathrm{input}}$ is set to 21.

In this study, a deep neural network is used to process the observation $o_{t}$, and its outputs are the selection probabilities of actions, denoted by $P(a_{t}|o_{t})$. The deep neural network architecture is shown in Figure 5.

As shown in Figure 5, we use four hidden layers to process the observation $o_{t}$. The first hidden layer uses the CNN to process the input data, which has 4 two-dimensional filters with kernel size = (2, 2) and stride = 1, and its activation function is ReLU [29]. The second and third hidden layers are two fully connected layers with 200 rectifier units. The last hidden layer contains nine nonlinear units with an activation function of Softmax, limiting the output to (0, 1), whose outputs are the selection probabilities of each action.

#### 3.3.4. Reward Function

The design of the reward function is closely related to our objective, which is to enable the UAV team to balance between giving the known targets a fair observation and exploring the search region. Thus, a reward function is designed to achieve this objective:

(28) $$r_{t}\left(u_{i}\right)=r_{t}^{1}\left(u_{i}\right)+r_{t}^{2}\left(u_{i}\right)+r_{t}^{3}\left(u_{i}\right)+r_{t}^{4}\left(u_{i}\right)$$

where $r_{t}\left(u_{i}\right)$ is the reward received by UAV $u_{i}$ at time step *t*, which is a sum of four different rewards.

The reward $r_{t}^{1}\left(u_{i}\right)$ encourages UAV $u_{i}$ to track targets that have been discovered, which consists of the following three terms:

(29) $$r_{t}^{1}\left(u_{i}\right)=\lambda_{1}{}^{l}r_{t}^{1}\left(u_{i}\right)+\lambda_{2}{}^{g}r_{t}^{1}\left(u_{i}\right)+\lambda_{3}{}^{h}r_{t}^{1}\left(u_{i}\right)$$

where ${}^{l}r_{t}^{1}\left(u_{i}\right)$ represents the local reward for tracking the discovered targets, ${}^{g}r_{t}^{1}\left(u_{i}\right)$ represents the global reward for tracking the discovered targets, ${}^{h}r_{t}^{1}\left(u_{i}\right)$ represents the reward for recording the historical positions of the targets, $\lambda_{1}$, and $\lambda_{2}$ and $\lambda_{3}$ are the positive coefficients. The rewards ${}^{l}r_{t}^{1}\left(u_{i}\right)$, ${}^{g}r_{t}^{1}\left(u_{i}\right)$ and ${}^{h}r_{t}^{1}\left(u_{i}\right)$ are designed as follows:

(30) $$\left\{\begin{array}{c} {}^{l}r_{t}^{1}\left(u_{i}\right)=\min(\sum_{j=1}^{N}\frac{d_{s}}{20*d_{t}\left(u_{i},\;\nu_{j}\right)},\;1),\;\mathrm{for}\;d_{t}\left(u_{i},\nu_{j}\right)<d_{s}\\ {}^{g}r_{t}^{1}\left(u_{i}\right)={\bar{\eta }}_{t}-{\bar{\eta }}_{t-1}\\ {}^{h}r_{t}^{1}\left(u_{i}\right)=\min\left(\mathrm{sum}\left(MT_{t}\left(u_{i}\right)\right),1\right) \end{array}\right.$$

where $d_{t}\left(u_{i},\nu_{j}\right)$ represents the distance between UAV $u_{i}$ and target $\nu_{j}$ at time step *t*, ${\bar{\eta }}_{t}$ represents the average observation rate of targets at time step *t*, $\mathrm{sum}(MT_{t}\left(u_{i}\right))$ represents the sum of the values of the elements in matrix $MT_{t}\left(u_{i}\right)$, min(x,y) returns the minimum value of *x* and *y*.

The reward $r_{t}^{2}\left(u_{i}\right)$ encourages UAV $u_{i}$ to explore the search region, which consists of the following two terms:

(31) $$r_{t}^{2}\left(u_{i}\right)=\lambda_{4}{}^{l}r_{t}^{2}\left(u_{i}\right)+\lambda_{5}{}^{g}r_{t}^{2}\left(u_{i}\right)$$

where ${}^{l}r_{t}^{2}\left(u_{i}\right)$ is the local reward for exploring the search region, ${}^{g}r_{t}^{2}\left(u_{i}\right)$ is the global reward for exploring the search region, $\lambda_{4}$ and $\lambda_{5}$ are the positive coefficients. The rewards ${}^{l}r_{t}^{2}\left(u_{i}\right)$ and ${}^{g}r_{t}^{2}\left(u_{i}\right)$ are designed as follows:

(32) $$\left\{\begin{array}{c} {}^{l}r_{t}^{2}\left(u_{i}\right)=\min\left(\frac{C_{L}C_{W}\left(\beta_{t}\left(u_{i}\right)-\beta_{t-1}\left(u_{i}\right)\right)}{M*d_{s}},\;1\right)\\ {}^{g}r_{t}^{2}\left(u_{i}\right)=\min\left(\frac{C_{L}C_{W}\left(\beta_{t}-\beta_{t-1}\right)}{M*d_{s}},\;1\right) \end{array}\right.$$

where $\beta_{t}\left(u_{i}\right)$ represents the local exploration rate of the search region known by UAV $u_{i}$ at time step *t*, $\beta_{t}$ represents the global exploration rate of the search region at time step *t*. $\beta_{t}\left(u_{i}\right)$ and $\beta_{t}$ are calculated as follows:

(33) $$\left\{\begin{array}{c} \beta_{t}\left(u_{i}\right)=\frac{1}{t}\frac{1}{C_{L}C_{W}}\sum_{k=1}^{C_{L}}\sum_{l=1}^{C_{W}}mc_{t}^{kl}\left(u_{i}\right)\\ \beta_{t}=\frac{1}{t}\frac{1}{C_{L}C_{W}}\sum_{k=1}^{C_{L}}\sum_{l=1}^{C_{W}}t_{stamp}\left(c_{kl}\right) \end{array}\right.$$

The reward $r_{t}^{3}\left(u_{i}\right)$ penalizes UAV $u_{i}$ for approaching other UAVs too close, which is designed as follows:

(34) $$r_{t}^{3}\left(u_{i}\right)=\sum_{j=1,j\neq i}^{M}r_{t}^{3}\left(u_{i},\;u_{j}\right),\;r_{t}^{3}\left(u_{i},\;u_{j}\right)=\left\{\begin{array}{c} -0.2,\;\mathrm{if}\;0.8d_{s}\leq d_{t}\left(u_{i},\;u_{j}\right)<d_{s}\\ -0.5,\;\mathrm{else}\;\mathrm{if}\;0.5d_{s}\leq d_{t}\left(u_{i},\;u_{j}\right)<0.8d_{s}\\ -1.0,\;\mathrm{else}\;\mathrm{if}\;d_{t}\left(u_{i},\;u_{j}\right)<0.5d_{s}\\ 0.0,\;\mathrm{else} \end{array}\right.$$

The reward $r_{t}^{4}\left(u_{i}\right)$ penalizes UAV $u_{i}$ for leaving the search region, which is designed as follows:

(35) $$r_{t}^{4}\left(u_{i}\right)=\left\{\begin{array}{c} -5,\;\mathrm{if}\;c_{t}\left(u_{i}\right)\notin S\\ 0,\;\mathrm{else} \end{array}\right.$$

The reward function designed above can make UAVs receive dense rewards in the training process, which can reduce the difficulty of learning. In addition, we set $\lambda_{1}=0.6$, $\lambda_{2}=0.2$, $\lambda_{3}=0.2$, $\lambda_{4}=0.7$, and $\lambda_{5}=0.3$ in the training process.

#### 3.3.5. Training Algorithm

In this study, we used a policy-based DRL algorithm, proximal policy optimization (PPO) [30], to train the deep neural network. The PPO has the benefits of optimizing control policies with guaranteed monotonic improvement and high sampling efficiency, and it has been widely used in the control of robots [31,32].

The algorithm flow is shown in Algorithm 1. In the training process, a centralized training, decentralized execution paradigm is used. Specifically, at each time step, each UAV independently obtains the observation and selects action through the shared policy, and the policy is trained with experiences collected by all UAVs during network training. The collected experience is used to construct the loss function $L^{CLIP}\left(\theta \right)$ for the policy network $\pi_{\theta }$ and the loss function $L^{V}\left(\phi \right)$ for the value network $V_{\phi }$. The value network structure is the same as the policy network structure, except that it has only one linear unit in its last layer. In each episode, the policy network $\pi_{\theta }$ is optimized $E_{\pi }$ times, and the value network $V_{\phi }$ is optimized $E_{V}$ times on the same minibatch data sampled from the collected experience with Adam optimizer [33].

## 4. Results

In this section, simulation experiments are performed to evaluate the effectiveness of our proposed policy. We first describe the simulation setup and introduce the training process. Then, we compare our policy with other methods in various scenarios to validate its performance. Finally, we discuss the results.

### 4.1. Simulation Setup and Training Results

We conduct simulation experiments in a Python environment. The deep neural networks are implemented with Pytorch [34]. In the training process, we consider a search region of size 50×50 cells, i.e., $C_{L}=C_{W}=50\;\mathrm{cells}$. The numbers of UAVs and targets in the search region are set to M=5 and N=10, respectively. The sensing range of each UAV is set to $d_{s}=5\;\mathrm{cells}$ and the communication range of each UAV is set to $d_{c}=10\;\mathrm{cells}$. In addition, the maximum UAV speed is set to 1 cell per time step, and the maximum target speed is set to 0.5 cells per time step. The total mission time step is 200, i.e., T=200. We set $t_{U}=5$ and $t_{T}=8$ for the decay period of the position history map of the UAVs and that of the position history map of the targets, respectively. The parameters in Algorithm 1 are listed in Table 1. In addition, the observation input size is set to $C_{\mathrm{input}}=\;21\;\mathrm{cells}$.
**Algorithm 1:** PPO with multiple UAVs for CMUOMMT
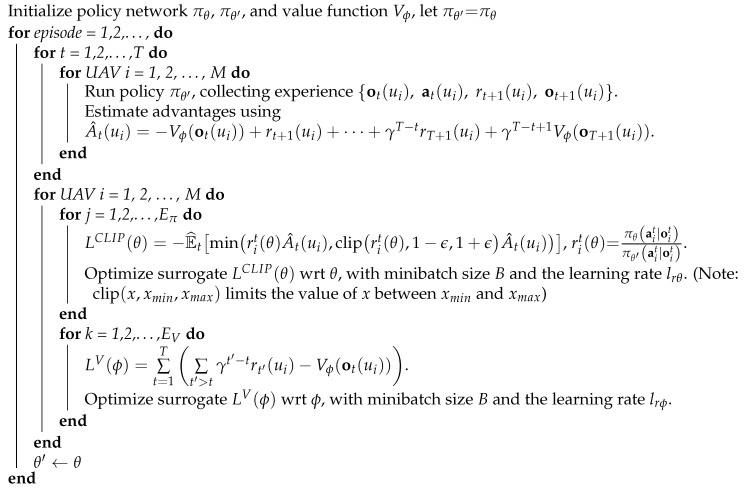


**Figure 6:**
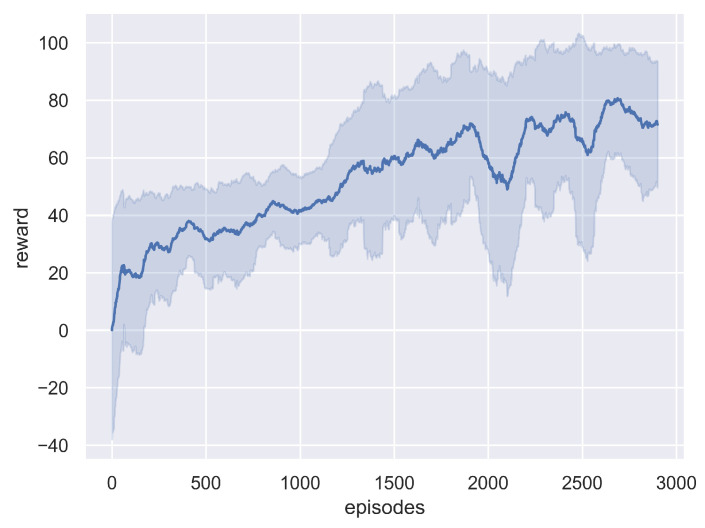
Training curve of the average and variance of each episode’s cumulative reward every 100 episodes.

The training process took 3000 episodes. At the beginning of each training episode, the positions of the UAVs and the targets are randomly reset. The speed of each target is randomly generated between [0, 0.5] cells per time step and remains unchanged during a training episode. We recorded the average and variance of each episode’s cumulative reward every 100 episodes. The cumulative reward for each training episode is the average of the cumulative rewards received by all UAVs. The training results are shown in Figure 6. As training progresses, each UAV receives progressively larger rewards, which means that the control policy gradually converges, allowing each UAV to track discovered targets and explore unknown environments. It is worth noting that in the early stages of training, the UAVs receive negative rewards due to leaving the search region.

### 4.2. Comparison with Other Methods

In this subsection, we compare our policy with other methods, including A-CMOMMT [6], P-CMOMMT [8], PAMTS [19], and Random policy. A-CMOMMT is a traditional approach for solving the CMOMMT problem, which uses weighted local force vectors to control UAVs. P-CMOMMT considers the uniformity of the observation of the targets compared with A-CMOMMT. PAMTS is a novel distributed algorithm, considering tracking the targets and exploring the environment in a unified framework. Random policy serves as a baseline of the CMOMMT problem.

In each set of comparative simulation experiments, we ran 50 random test experiments for each method and calculated the average of the following three metrics:

- the average observation rate of the targets $\bar{\eta }$,
- the standard deviation $\sigma_{\eta }$ of the observation rates of the *N* targets, and
- the exploration rate β of the search region.

**Figure 7:**
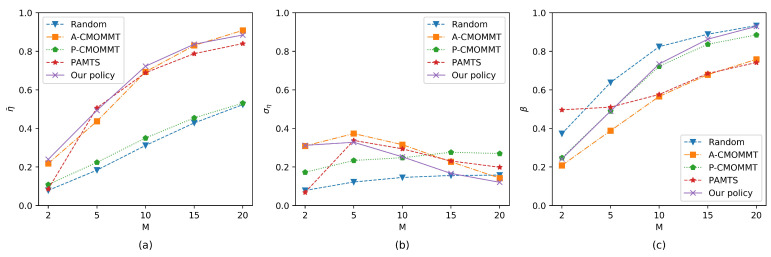
Comparison of results when the number of UAVs is increasing while the number of targets is fixed to 10. Other parameters are the same as those in the training process. (**a**) The results of the average observation rates change with the number of UAVs. (**b**) The results of the standard deviation of the average observation rates change with the number of UAVs. (**c**) The results of the exploration rates change with the number of UAVs.

We first compared our policy against other methods with different numbers of UAVs while the number of targets was fixed to 10. As shown in Figure 7a, the average observation rates of the targets continued to increase as the number of UAVs increased across all methods. Our policy had the best performance when the number of UAVs was 2, 10, or 15, and it was the second best method when the number of UAVs was 5 or 20. In addition, Figure 7b shows that our policy had the minimum standard deviation of the observation rates compared with A-CMOMMT and PAMTS in most cases, which shows that our policy can give the targets relatively fair observations. It is worth noting that the standard deviation of the observation rates gradually increased with the increase in the number of UAVs when using P-CMOMMT and Random policy. This is because the number of targets being observed increases when the number of UAVs increases, so that the standard deviation of the observation rates also increases with it. Figure 7c shows the exploration rate of the search region with the various number of UAVs. It can be seen that our policy had a high exploration rate in most cases relative to other methods except for the random policy. Overall, our policy can give targets a high and fair observation while maintaining a high exploration rate of the search region.

**Figure 8:**
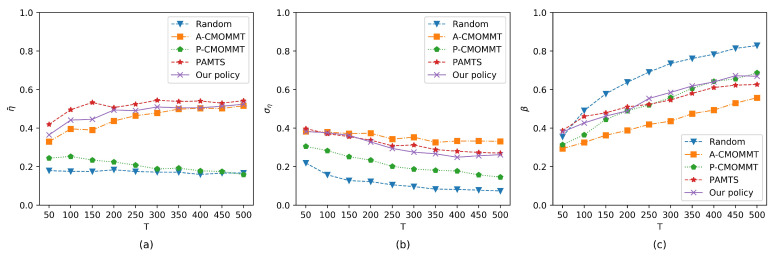
Comparison of results when the total mission time is increasing. Other parameters are the same as those in the training process. (**a**) The results of the average observation rates change with the total mission time. (**b**) The results of the standard deviation of the average observation rates change with the total mission time. (**c**) The results of the exploration rates change with the total mission time.

The impact of the total mission time on the three metrics was also studied. Figure 8a shows that the observation rates with A-CMOMMT, PAMTS, and our policy continued to improve as the total mission time increased. It is because that the increased mission time allows UAVs to search the environment sufficiently to find the targets. In addition, the observation rates of the A-CMOMMT, PAMTS, and our policy gradually approached as the total mission time increased, which means all three methods can find the targets in the search region with enough mission time. P-CMOMMT had a low observation rate because it tries to give a uniform observation to the targets, which can also be seen from Figure 8b, where P-CMOMMT had a relatively low standard deviation of the observation rates. As shown in Figure 8b, our policy had a medium standard deviation of the observation rates. Similarly, as shown in Figure 8c, our policy had a medium exploration rate compared to the other methods. The results show that our policy can increase the observation rate of the targets when the mission time increases, while reducing the standard deviation of the observation rates and increasing the exploration rate of the search region.

**Figure 9:**
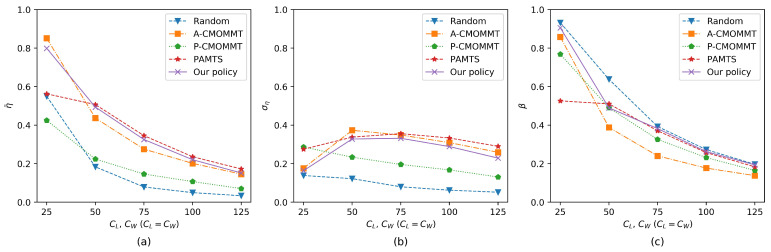
Comparison of results when the size of the search region is increasing. Other parameters are the same as those in the training process. (**a**) The results of the average observation rates change with the size of the search region. (**b**) The results of the standard deviation of the average observation rates change with the size of the search region. (**c**) The results of the exploration rates change with the size of the search region.

In addition, the impact of the size of the search region on the three metrics is studied. Figure 9a,c shows that the observation rate of the targets and the exploration rate of the search region decreased as the size of the search region increased. It is obvious that targets were more scattered in a larger search region, which makes it difficult for UAVs to find targets and explore the entire search region. As shown in Figure 9b, the increase in the standard deviation of the observation rates from $C_{L}=C_{W}=25$ to $C_{L}=C_{W}=50$ was due to the number of the discovered targets decreasing as the size of the search region increased. However, the decrease in the standard deviation of the observation rates from $C_{L}=C_{W}=50$ to $C_{L}=C_{W}=125$ was due to the difficulty for UAVs to find the targets in a large search region.

**Figure 10:**
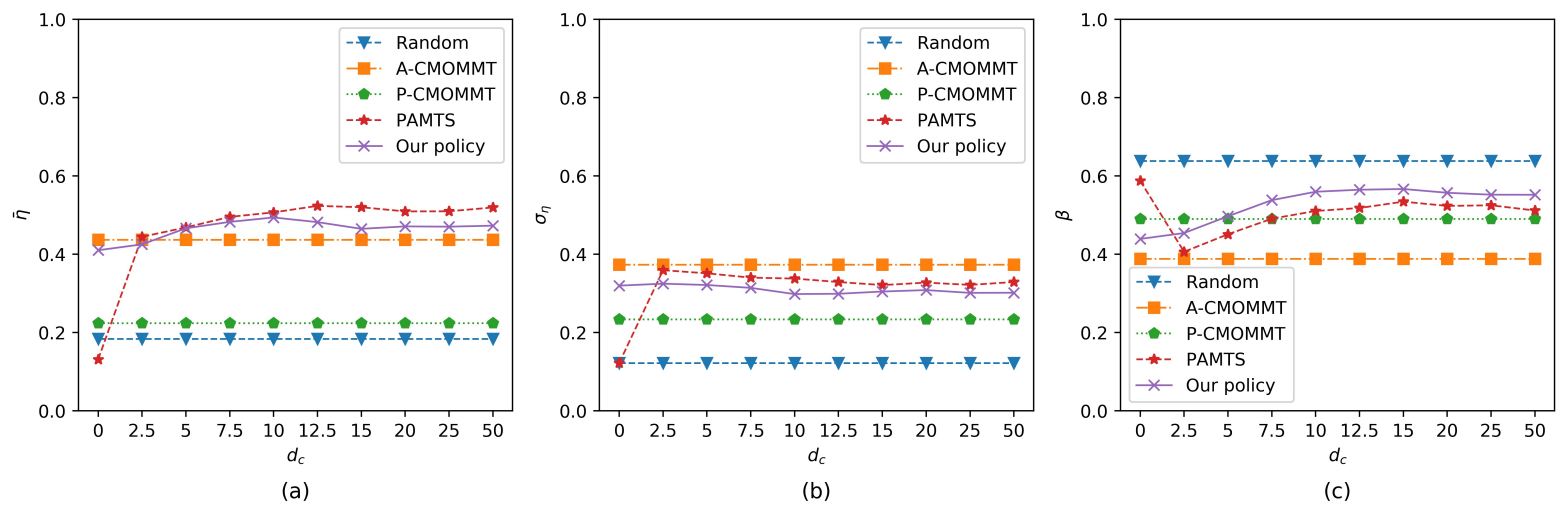
Comparison of results when the communication range is increasing. Other parameters are the same as those in the training process. (**a**) The results of the average observation rates change with the communication range. (**b**) The results of the standard deviation of the average observation rates change with the communication range. (**c**) The results of the exploration rates change with the communication range.

Finally, we studied the impact of the communication range on the three metrics. As shown in Figure 10, for our policy, the observation rate and the exploration rate continued to improve, and the standard deviation of the observation rate continued to decrease as the communication range increased, until the communication range was greater than 10 cells, where all three metrics basically no longer changed. The impact of the communication range on the three metrics under PAMTS was consistent with our policy, except when there was no communication among UAVs, i.e., $d_{c}=0$ cells. The results show that the information from the nearby UAVs can bring significant improvements, and the information from the remote UAVs has little impact on this mission. In addition, because A-CMOMMT and P-CMOMMT only consider the impact of UAVs within the sensing range, the variation in communication range has no effect on the three metrics. Like the above results, our policy has a high observation rate just below PAMTS and a low standard deviation of the observation rates and a high exploration rate of the search region compared with A-CMOMMT and PAMTS.

### 4.3. Discussion

The above comparison results show that our policy can find a balance between giving the known targets a fair observation and exploring the search region. Though our policy has a low observation rate compared with PAMTS in most cases, it can give a fair observation to the targets with a low standard deviation of the observation rates and continue a high exploration rate of the search region, which can enable UAVs to find more targets when the total number of the targets is unknown. It is worth noting that PAMTS assumes that the total number of targets is known, and we do not have this assumption.

## 5. Conclusions

In this paper, a DRL based approach is proposed to solve the CMUOMMT problem. Unlike traditional CMOMMT approaches, we considered the average observation rate of the targets, the standard deviation of the observation rates, and the exploration rate of the search region at the same time under the assumption that the total number of the targets is unknown. To achieve this objective, we used four observation maps to record the historical positions of targets and other UAVs, exploration status of the search region, and the UAV’s position relative to the search region. In addition, UAVs’ maps were merged from the maps of different UAVs within a communication range. The merged maps were then cropped and processed with a deep neural network to obtain the selection probabilities of actions. The reward function was designed carefully to provide UAVs with dense rewards in the training process. The results of the extensive comparison simulation experiments prove that our policy can give the targets a fair observation and meanwhile maintain a high exploration rate of the search region. Future work will study the CMUOMMT problem in a search region with obstacles and targets with evasive movements. This is a more challenging problem that requires smarter collaboration between UAVs.

## Figures and Tables

**Table 1:** Training parameters in Algorithm 1.

Parameters	Values
*T*	200
*M*	5
γ	0.99
$E_{\pi }$	10
ε	0.1
*B*	64
$l_{r\theta }$	0.00005
$E_{V}$	10
$l_{r\phi }$	0.001

## Data Availability

No new data were created or analyzed in this study. Data sharing is not applicable to this article.

## Abbreviations

The following abbreviations are used in this manuscript:

A3C	Asynchronous advantage actor-critic
CMOMMT	Cooperative Multi-Robot Observation of Multiple Moving Targets
CMUOMMT	Cooperative Multi-UAV Observation of Multiple Moving Targets
CNN	Convolutional neural network
DRL	Deep reinforcement learning
EKF	Extended Kalman filter
FOV	Field of view
GA	Genetic algorithm
MOSOS	Multi-Objective Symbiotic Organisms Search
MPC	Model Predictive Control
PHD	Probability Hypothesis Density
POMDP	Partially Observable Markov Decision Process
PPO	Proximal policy optimization
PSO	Particle Swarm Optimization
SAR	Search and rescue
UAV	Unmanned aerial vehicle
